# Framing Facets of Social Participation: Older Adults’ Experiences of “Social Online Meetings”

**DOI:** 10.1177/15394492241262291

**Published:** 2024-06-24

**Authors:** Maria Löfgren, Anneli Nyman, Gunilla Isaksson, Ellinor Larsson

**Affiliations:** 1 Luleå University of Technology, Sweden

**Keywords:** older adults, social participation, grounded theory

## Abstract

Changing demographics with an increased proportion of older adults indicate the need to develop new health-promoting interventions where the potential of digitization is considered. The aim was to explore and create an understanding of how social online meetings are experienced by older adults. Interviews with older adults generated data that were analyzed using a grounded theory approach. The participants were interviewed after participating in a digital health promotion group initiative provided in a municipality context. A core conceptual category and three subcategories reflected an intertwined process of discovering facets of social participation where internal reflections on personal values and needs were nurtured by an external driven process of becoming part of a group in an online context. Occupational therapists and other health and social care professionals need to consider the various facets of social participation when supporting older adults active and healthy aging.

## Plain Language Summary

Older Adults’ Experiences of “Social Online Meetings”

Promoting health among older adults is an important societal issue. Changing demographics with an increased proportion of older adults indicate the need to develop new health-promoting interventions. However, it is important to explore how such interventions in municipal settings meet the needs of older adults and the circumstances that influence their participation. Therefore, this study aimed to understand how older adults experience social online meetings. Seven older adults who had participated in an intervention called “social online meetings” were interviewed about their experiences. The findings demonstrate how older adults come to explore various dimensions of social participation and the value it holds for their everyday life. This gave rise to achieving new perspectives on everyday activities and encouraged personal development, and generated ideas for change. Occupational therapists and other health and social care professionals should consider how older adults’ social participation can be promoted when designing health-promoting interventions online.

## Introduction

In Sweden, health care services are transforming from reactive to proactive service delivery where health-promoting efforts are emphasized and citizens are expected to take greater responsibility for their health (Swedish Government Official Reports, 2018; 2020). In addition, changing demographics with an increased proportion of older adults (World Health Organization [WHO], 2017) indicate the need to develop new health-promoting interventions. Earlier health-promoting interventions have shown promising results in enhancing health outcomes and well-being among older adults (Bajraktari et al., 2020; Zingmark et al., 2014). The focus of these interventions is diverse but often includes components such as fall prevention, physical activity, and nutrition (Bajraktari et al., 2020). The interventions can also include health education and social aspects and can be provided in a group format (Bajraktari et al., 2020; Duplaga et al., 2016).

Health-promoting interventions in group formats for older adults that include social aspects can offer opportunities for social support (Behm et al., 2016; Gustafsson et al., 2017), the opportunity to be acknowledged, and enhanced feelings of belonging to a community (Lapena et al., 2020), as well as relieving experiences of loneliness or social isolation (Fakoya et al., 2020; Tong et al., 2021). Although social aspects are included in interventions, the primary focus is rarely on social participation (Turcotte et al., 2018).

Social participation is important for experiences of health and well-being among older adults (Holt-Lunstad et al., 2015; Levasseur, Desrosiers, & Whiteneck, 2010) and can be described at different levels of engagement (Levasseur, Richard, et al., 2010). It includes involvement in everyday activities, providing interactions with others in community life and important shared spaces based on what individuals want and is meaningful to them (Levasseur et al., 2022). However, social and environmental circumstances, such as fading social relations and changing social venues (Löfgren et al., 2021), often impact older adults’ access to important everyday activities, resulting in fewer opportunities for social participation (Calder et al., 2018; Townsend et al., 2021). Therefore, it is essential to focus on social participation in health-promoting interventions.

To increase the availability and delivery of services, the potential of digitalization needs to be considered when developing health-promoting interventions in health and social care (Koh et al., 2021; Swedish Government Official Reports, 2019). Furthermore, it is important to ensure older adults’ opportunities for digital inclusion in society (Steinman et al., 2023; Xie et al., 2020). Previous research on health-promoting interventions in online group formats has shown potential for improving the social participation and well-being of older adults (Baker et al., 2018; Poscia et al., 2018; Shapira et al., 2021). However, there is a need to further explore how digital tools and online delivery can be utilized to promote social participation among older adults (Chen & Schulz, 2016; Larsson et al., 2020).

Current health-promoting interventions for older adults in municipal contexts are delivered by various professionals, including occupational therapists; they can be provided in different forms, for example, by physical or digital meetings, for individuals or groups (Bajraktari et al., 2020; Norberg et al., 2021). However, there is a lack of knowledge regarding older adults’ experiences with such interventions provided in a municipality context (Bajraktari et al., 2020). Previous research has also highlighted the need to explore the circumstances and contexts of promotive interventions for older adults (Fakoya et al., 2020). There is a need to know more about how health-promoting group interventions delivered in online contexts are experienced by older adults and whether such interventions can promote social participation. In addition, it is important to understand how interaction and engagement occur within a group in an online context. Such knowledge could provide insights into how health-promoting interventions can be developed further within municipality contexts to support older adults’ social participation. Therefore, this study aimed to explore and create an understanding of how social online meetings are experienced by older adults.

## Method

### Study Design

The study drew on the constructivist grounded theory approach (Charmaz, 2014) to guide the data generation and analysis. This approach was chosen because the research was designed to study the actions, social situations, and processes that occur in everyday life situations, that is, experiences of participating in the digital health promotion group initiative, hereafter referred to as “social online meetings.” The purpose was to discover patterns that emerge in the data and to inductively formulate an abstract understanding of the studied experiences.

### Study Context

This study was conducted in a municipality in northern Sweden, where social online meetings were offered to older adults within a municipality-based health promotion service. The social online meetings consisted of a period of eight meetings and were offered to a group of four to six older adults on one or two occasions per semester. The meetings covered different topics and lasted approximately 1 hr. Diverse professionals were responsible as group leader for the different meetings, while one meeting was arranged by the older adults themselves. The period of meetings was coordinated by an occupational therapist or assistant nurse at the municipality-based service. Table 1 provides a summary of social online meetings.

**Table 1.** Overview of the Social Online Meetings Provided in the Municipality-Based Service.

Group meeting	Content of meeting	Group leader
Week 1	Introduction and presentation of participants	Occupational therapist or Assistant nurse
Week 2	Digital tools, online activities and library services	Librarian
Week 3	Physical activity	Physiotherapist
Week 4	Everyday activities and technical aids	Occupational therapist
Week 5	Municipality-based services for older adults	Social worker
Week 6	Nutrition and eating habits	Nurse
Week 7	Social meeting arranged by the participants	No group leader
Week 8	Completion and summary of the initiative	Occupational therapist or Assistant nurse

### Sampling and Participants

Purposive sampling (Holloway & Galvin, 2017) was used to recruit participants. An information letter was sent to the older adults by the coordinator after they had participated in a period of social online meetings at the time of recruitment (from spring 2021 until spring 2022). The information letter was only sent to the older adults who had attended more than one meeting. The letter included information about the study, that participation was voluntary, and how to contact the first author to gain more information. A total of 11 letters were sent, in response to which seven older adults provided written informed consent to participate. See table 2 for participants characteristics.

**Table 2.** Participants Characteristics.

Participants	
Total number	7
Sex	
Women	4
Men	3
Age	
Range	66–87
*M*	78
Social status	
Living alone	6
Cohabiting	1
Accommodations	
House	2
Apartment	5
Education	
Upper secondary school	3
University/university college education	4
Number of meetings attended	
All meetings	3
6–7 meetings	2
4–5 meetings	1
Uncertain	1

### Data Generation and Analysis

Data were generated using qualitative interviews. The location of the interviews was determined in dialogue with the participants. Initially, seven interviews were conducted. Four were conducted as physical meetings at the participants’ homes, gardens, or outside at a location selected by the participants. The remaining three were conducted as video meetings. All interviews were digitally recorded and lasted between approximately 30 and 70 min. An interview guide based on open-ended questions was used, and participants were encouraged to narrate their experiences related to their participation in the social online meetings. Examples of the questions were as follows: *describe the impact that attending social online meetings has had on you*, *and describe how the social online meetings have influenced your daily life*. In accordance with grounded theory (Charmaz, 2014), data generation and analysis were conducted iteratively; information from early interviews was used to improve the later interviews.

The first author transcribed all the interviews verbatim. The initial interviews were coded segment by segment with a focus on participants’ experiences of their participation in the social online meetings and its relevance for their everyday lives. The codes that emerged remained close to the data, for example, *making new acquaintances* and *becoming receptive to new things.* The initial codes were assembled and tentatively labeled with headings by the first author. Hence, data generation was developed successively based on ongoing analysis. Gradually, more interviews were coded, and new codes were compared with previous codes. Then, all the authors met to discuss the content and find a common understanding. During these analytical discussions, tentative categories started to emerge from the focused coding, such as *revealing and evaluating my everyday life* and *developing prospects for the future*. At this stage, follow-up interviews were planned to obtain more comprehensive data and enhance the evolving categories. Six participants provided rich data during the first interview and were invited to participate in a follow-up interview, three of whom agreed to participate. These interviews took place as physical meetings.

Throughout the process of data generation and analysis, memos (Charmaz, 2014) were written that explained and expanded upon the emerging categories. All the authors discussed the coding and compared the emerging categories regularly. To enhance the credibility of the interpretations, a back-and-forth technique (Lincoln & Guba, 1985) was employed to check emerging findings with data. The analysis remained closely focused on the data until late in the process of creating trustworthy categories grounded in the data. Consistent with the methodology (Charmaz, 2014), theoretical sampling was employed to further search for an abstract understanding of how social online meetings can be comprehended. At this point, the inductive analysis was further understood by employing a theoretical perspective on social participation (Levasseur et al., 2022).

### Methodological Considerations

Credibility was ensured by following and describing constructivist methodological procedures for data collection, coding, and theoretical sampling (Charmaz, 2014). Consequently, all the authors discussed and participated in the proceeding analysis, providing an opportunity to challenge preconceptions, and allowing different explanations to emerge. The involvement of multiple authors posing analytical questions in the analysis served to enhance the trustworthiness (Lincoln & Guba, 1985). Furthermore, the evolving results were discussed with the participants in the second interview. Constant comparison facilitated the authentication of findings, as this approach ensured that the analysis was grounded in the data.

## Findings

**Figure 1.**
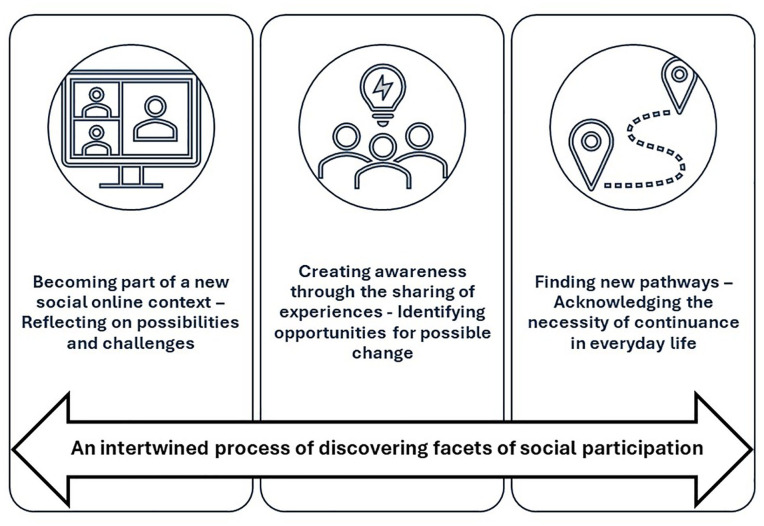
The Intertwined Process of Discovering Facets of Social Participation.

Based on the analysis, a core conceptual category and three subcategories were developed. The core conceptual category addresses the central features of all categories and illustrates the intertwined processes associated with participating in social online meetings. The three subcategories describe different aspects related to the core conceptual category. Overall, these findings can improve our understanding of how facets of social participation are reflected in participants’ experiences. Figure 1 illustrates the findings.

### An Intertwined Process of Discovering Facets of Social Participation

This core category reflects how engaging in social online meetings directed the participants’ focus to explore various facets of social participation and its value for their everyday life. Discovering social participation in interactions with others was comprehended as an intertwined process, including explorations of personal values and needs, which were nurtured by the externally driven process of becoming part of a group and interacting with others. Being part of this new social online context created opportunities for the participants to consider their own experiences in relation to those of others and thereby support the discovery of additional perspectives on life. Moreover, by interacting with others in a group context, a sense of community was created and ways to create and maintain social participation were uncovered and further explored. Finding new pathways to be engaged on different levels, in different social activities and in new arenas in society, was reflected as important for maintaining and developing social participation. The complexity of various facets of social participation to support such engagement is reflected in the participants’ experiences in the following three subcategories.

#### Becoming Part of a New Social Online Context—Reflecting on Possibilities and Challenges

This subcategory illuminates the participants’ experiences during the time when they entered the social online meetings and how they retrospectively described being engaged in the meetings. Becoming part of the new online context was reflected not only as a positive experience of meeting others but also as a struggle to navigate challenges of using the online platform sufficiently.

The experiences of the participants reflected that they had not clearly conceptualized their expectations before entering the social online meetings. As the meetings progressed, they continuously assessed how the other participants, the content of the meetings, and the possibility of finding community could meet their evolving expectations. The participants reflected on how exploring the social online context became important in relation to their individual needs and prerequisites. The process and focus of becoming a part of the group was partly directed toward social aspects that had previously been restrained due to the COVID-19 pandemic and for personal reasons, such as difficulties leaving their home or caring for a spouse. When interacting with other participants in the group, the online social group contributed to uplifting and revitalizing positive feelings and made participants feel less lonely, as described by one participant:

It has been pretty depressing for me for some time. However, meeting and talking to other people has been invigorating. I usually feel quite lonely. (Participant 1)

While some participants acknowledged being revitalized, others were skeptical about whether participating in the online meetings could meet their social needs, which was partially dependent on the size of the groups. Some participants appreciated the safe and comfortable atmosphere that was created when the group was sufficiently small for everyone to have their voice heard. However, others reported having the opposite experiences, that is, that the group was too small and that their expectations of developing social contacts were not met.

Participants’ ability to interact and communicate with each other also depended on how well they were able to use the online platform during the meetings. Moreover, some participants experienced barriers to engaging in group conversations, as their expectations of more interpersonal interactions were not met, as illustrated in the following quote:

I think it is hard to get personal contact when it is all online. If you are sitting together in a group somewhere, it is different. Then, you can chat with the person sitting next to you, maybe ask, can we meet sometime? When it is online, it doesn’t turn out that way. (Participant 3)

Another difficulty with social online meetings was that other participants interfered with digital shortcomings, which could impede contact and communication, as exemplified in the following quote:

I got a bit annoyed. Some participants didn’t pay attention. It seemed like they weren’t interested . . . or maybe they had trouble with handling their computers. (Participant 6)

Furthermore, if participants invested a great deal of energy and concentration in the use of the online platform and technical devices during the meetings, this influenced their opportunities to connect with the group and their impressions of what participation could offer from a more long-term perspective.

#### Creating Awareness Through the Sharing of Experiences—Identifying Opportunities for Possible Change

This subcategory focuses on how participating in social online meetings became important in creating the opportunity to share experiences with others. Sharing experiences of everyday life made it possible to mirror one’s own experiences in light of others, which contributed to new insights and provided possibilities to reevaluate one’s own situation. As such, being part of the social online meetings provided participants with opportunities to examine their everyday lives by adopting alternative perspectives.

The participants reflected on how they directed their focus to their own personal doings by considering the information given from professionals in combination with sharing experiences with other participants. The participants also recognized behaviors and strategies that they employed to maintain their health, such as embracing social engagement opportunities and employing various exercise or dietary habits. In addition, sharing experiences with others inspired personal reflections on different situations in everyday life and reminded them of the knowledge they already possessed. For example, different opportunities to engage in social activities and to enter new arenas in society were highlighted, which could serve as a starting point for identifying new perspectives on the participants’ personal situations.

Sharing experiences with others also enabled participants to gain greater appreciation of their personal situation. Moreover, they became aware of things they could still achieve and how they made efforts in everyday life to promote personal well-being. In addition, sharing experiences with others and finding additional perspectives created awareness of the possibilities for exploring one’s personal needs and preferences and entailed a process of confirmation, as illustrated in the following quote:

Hearing others, you learn new things, and you’ll have something to compare to. You get a reminder that life isn’t that easy for any of us at times. . . And actually, I can do plenty. (Participant 4)

Reflecting on one’s own situation and considering new perspectives were described as satisfying by the participants. However, the participants’ experiences reflected the importance of paying attention during meetings, actively listening to each other, and contributing to the group. This could be perceived as demanding and required preparation and focus. They found the online context challenging in this regard, and the need for support from others emerged in various ways. Through the process of identifying their personal needs, participants’ experiences reflected an awareness of how external factors such as technical support and economic resources could influence their ability to participate in social online activities. The fact that others had similar difficulties revealed experiences of a more collective need for support. This situation was exemplified as follows by one of the participants:

In society, when it comes to initiatives like this, you need to have resources! That there is some available technical support. This needs to be solved better. (Participant 4)

#### Finding New Pathways—Acknowledging the Necessity of Continuance in Everyday Life

This subcategory covers participants’ reflections concerning continuance in everyday life after finishing their participation in social online meetings. It reflects their aspirations for the future and reasoning about both possibilities and challenges. Furthermore, when they summarize their perspective on engagement in social activities or new social arenas, insights ultimately obtained include the necessity of continuance in situations in everyday life where they want to uphold and develop social participation. However, participants also emphasize experiencing ambiguity regarding how to progress and which paths to take for the future.

Becoming part of the social online meetings challenged the participants to handle situations they perceived as demanding. Managing the new social situation in the online context and daring to think of oneself as capable of confronting new challenges were positive experiences. In addition, such engagement could contribute to the courage and inspiration necessary to take the leap to participate in other communities and contexts. As a result of this new confidence gained, some participants expressed how they were exploring new paths and were either planning to or had already integrated new activities into their everyday life, such as joining an organization. These activities provided meaningful experiences and contributed to personal growth, and were described as having a positive influence on perceived health.

Participants’ engagement in social online meetings increased their understanding of their needs and preferences for social participation by being engaged in social interactions with others. Participants reflected on how participating in social online meetings was not considered a finishing point; rather, it was described in terms of continuance toward developing and challenging oneself and seeking proper arenas for doing so. As exemplified by one of the participants in the following quote,

Without opportunities like these, I do not know how it would have turned out. I think I would have felt very bad anyway. . . You have to continue to make sure that you try to fill your life with what you enjoy and benefit from. (Participant 5)

Although participation could open up paths for the future, some participants remained indecisive about how to move forward. They faced obstacles related to finding communities, engaging in activities of personal choice, or receiving support to engage, as illustrated by the following quote:

Since the meetings terminated, I don’t truly know what to do. It seems that you will have to come up with things on your own. (Participant 7)

Moreover, some participants perceived the group as a beneficial initiative, yet expressed a need for its continuation, as illustrated by the following quote:

I thought this group was a good idea, but it would have been great to go on. It was a convenient way to make contacts and friends online. (Participant 2)

## Discussion

The findings of this study reflect how older adults can discover facets of social participation when engaging in social online meetings in a municipality context. Participating in the social online meetings was experienced as an intertwined process, entailing both possibilities and challenges. The engagement of the participants was largely influenced by the online context.

The findings show that while exploring facets of social participation (Levasseur et al., 2022), participants’ focus is directed toward the value that social participation can have, as participants come to reflect upon the positive aspects of different facets of social participation, that is, learning more of available options in society, the enjoyment of socializing with others, and developing by challenging oneself in a new online arena together with others. These facets of social participation could be seen as a reflection of its importance to their well-being, in line with previous research (Nivestam et al., 2023). Interactions with others in the group provided opportunities to reevaluate one’s own situation and explore new perspectives on everyday activities and social contacts, leading to new insights. In addition, the findings indicate how sharing experiences with others created awareness of possibilities to make changes in everyday life. These findings coincide with earlier findings emphasizing how engaging in group interventions can support older adults and enhance experiences of belonging (Lapena et al., 2020). However, the group could be perceived both as a promoter of and a barrier to the level of engagement. Hence, health promotion aimed at older adults should involve thorough consideration of the composition of the group (Norberg et al., 2021), as well as community-building aspects.

Moreover, to fully realize the potential of social online meetings, it is important to address the various challenges faced by older adults, that is, difficulties related to preparing for using the digital tools necessary for involvement in the meetings, participating in the new online context, and engaging with others. These challenges reflected in the findings were intertwined with the experiences of possibilities for social participation and embedded in a transactional interplay with environmental aspects such as how the online context is experienced, how societal structures impact participation, and individuals’ diverse preferences (Egan & Restall, 2022; Nivestam et al., 2023). Furthermore, these different aspects cannot be separated from each other, and they influence the overall experience of participation. Therefore, it is important to comprehensively approach the participation of older adults in social online meetings, as beneficial implementation may depend on several interacting aspects.

In addition, consistent with the findings of previous research (Steinman et al., 2023), older adults encountered difficulties associated with the use of technology and the online platform and required support in this regard. This finding is in line with previous findings showing how older adults continue to face restricted digital competence (Raja et al., 2023). This can be disempowering because it creates a digital divide (Köttl et al., 2021). To reduce this digital divide and ensure enhanced opportunities for active and healthy aging, awareness of and support for lifelong learning and inclusive technology design are necessary (Han & Nam, 2021; Köttl et al., 2021). In addition, financial resources, together with policies and practices that support digital competence, access, and usability for older adults, are important for supporting their inclusion in society (Steinman et al., 2023; Xie et al., 2020). If older adults are to actively promote their social participation and health (Swedish Government Official Reports, 2018; 2020), it is equally important to consider societal commitments, such as economic, social, and environmental policies. Therefore, this study, along with others, highlights the significance of societal decisions that help facilitate older adults in performing desired activities (Nivestam et al., 2023), including activities related to social participation.

The findings of this study show the potential to support and develop social participation through health promotion social online meetings. However, challenges related to the digital context and opportunities to engage with the group can be barriers. Thus, it is important for occupational therapists together with other health and social care professionals to further involve older adults in how group interventions supporting active and healthy aging can be developed (Chagnon et al., 2023) in, for example, online settings. To develop and provide interventions for social participation among older adults, occupational therapists can utilize their professional competence by considering individual circumstances and needs. Potentially, the group online setting could provide alternative and adaptable ways to explore the facets of social participation with others.

The findings illustrate how participation in social online meetings offers older adults’ opportunities to explore everyday activities that can promote health and well-being. This is in line with earlier findings showing that participation in group format interventions may function as a catalyst for personal development and to create meaning (Nilsson & Lundgren, 2018). By facilitating opportunities to share experiences with others and to find additional perspectives in everyday life, the group format can hold potential in interventions in different contexts. In line with recent occupational therapy theory (Egan & Restall, 2022), occupational therapists are challenged to develop their practice with different groups in societal contexts. Moreover, supporting older adults’ interactions in unfamiliar settings, utilizing digital tools, and facilitating groups with diverse requirements could make interventions even more valuable for promoting social participation. The findings indicate that enabling older adults to develop and implement changes in everyday activities to support social participation is important, and future research could further consider older adults’ experiences using digital tools for promoting social participation.

## Conclusion

This study revealed that older adults’ experiences with participating in social online meetings encompass various facets of social participation, reflected as an intertwined process. Finding prospects and discovering facets of social participation gave rise to opportunities for achieving additional perspectives and encouraged further engagement and ideas for change in everyday life. Furthermore, continuance was perceived as important for maintaining social participation and experiences of health and well-being. In light of these findings, occupational therapists and other health and social care professionals should consider how older adults’ social participation can be promoted when designing health promotion group interventions online. Future research can explore how various facets of older adults’ social participation can be used in different health promotion initiatives. In addition, future studies could aim to improve our understanding of how to develop societal efforts to facilitate older adults’ social participation.
